# Activity of Bradykinin B_2_ Receptor Is Regulated by Long-Chain Polyunsaturated Fatty Acids

**DOI:** 10.1371/journal.pone.0068151

**Published:** 2013-06-27

**Authors:** Jose Candelario, Mirianas Chachisvilis

**Affiliations:** La Jolla Bioengineering Institute, San Diego, California, United States of America; UAE University, Faculty of Medicine & Health Sciences, United Arab Emirates

## Abstract

The molecular and cellular mechanisms by which long-chain polyunsaturated fatty acids (LCPUFA) exert their beneficial effects on cardiovascular health remain obscure. While both LCPUFA and bradykinin (BK) signaling pathway play a role in the cardiovascular system, any direct link between the two is yet to be established. Using picosecond time-resolved fluorescence microscopy and a genetically engineered bradykinin B_2_ receptor (B_2_R) sensor (B2K-CC), we detected LCPUFA-induced conformational responses in the B_2_R similar to those caused by its cognate ligand, BK. The selective B_2_R antagonist (HOE-140) blocked the eicosapentaenoic acid (EPA, C20∶5, n-3) induced conformational response of the B2K-CC. Further analysis suggests that LCPUFA are capable of direct, B_2_R-dependent activation of extracellular ligand-regulated kinases (ERK). From a wide range of fatty acids studied, varying in chain length, saturation, and position of double bonds, EPA, docosahexaenoic (DHA, C22∶6, n-3), docosadienoic (DDA, C22∶2, n-6), and dihomo-gamma linoleic (DGLA, C20∶3, n-6) fatty acids caused the highest ERK phosphorylation. EPA or DHA dependent ERK phosphorylation was inhibited by the selective B_2_R antagonist. We show that LCPUFA stimulates downstream signaling by B_2_R such as B_2_R-dependent phosphorylation and expression regulation of endothelial nitric-oxide synthase (eNOS). Further analysis indicated that LCPUFA also alters levels of the eNOS transcription factor, kruppel-like factor 2 (KLF2). Moreover we show that EPA increases membrane fluidity on the same time scale as B_2_R conformational response, suggesting that partitioning of LCPUFA into bilayer is a primary step required for receptor activation. In summary our data show that LCPUFA activate B_2_R receptor at nanomolar concentrations suggesting a novel molecular mechanism by which fatty acids may affect the cardiovascular system.

## Introduction

We have recently reported that LCPUFAs stimulate conformational changes and activate parathyroid hormone receptor (PTH1R) in a dose dependent manner [1]. PTH1R is a G protein coupled receptor (GPCR) involved in bone homeostasis [2]. There has been increasing consensus that many GPCRs are “allosteric machines” controlled by various membrane components such as lipids, ions, cholesterol or mechanical perturbation of the cell membrane [3]–[10]. For example, we have shown that bradykinin B_2_R (a GPCR expressed in endothelial cells [11]) responds to mechanical perturbation and changes in fluidity of the cell membrane [12]. B_2_R activates multiple signaling cascades but generally is reported to signal through G_q_-protein - phospholipase C pathway stimulating phosphoinositide hydrolysis and transient increase in intracellular free Ca^2+^. B_2_R also signals through the G_s_-cAMP-protein kinase A and mitogen-activated protein kinases (MAPKs) leading to various biological effects [11]. B_2_R signaling pathway is involved in activation of eNOS, the enzyme that catalyze the production of nitric oxide (NO) [13], which is essential in maintaining normal vessel homeostasis [14]. The coronary dilation induced by bradykinin is mediated by the activation of the B_2_R. A number of earlier studies have demonstrated that omega-3 polyunsaturated fatty acids induce in-vivo vasodilation [15]–[20]. These observations along with established effects of fatty acids on cardiovascular health provide a strong rationale for the study of the signal transduction pathway of LCPUFAs in endothelial cells. Since endothelial cells are exposed to fatty acids at high concentrations, in this study we sought to determine if LCPUFA can also regulate function of the B_2_R. The venous blood concentration of fatty acids is known to vary from 250 µM to 3 mM depending on the nutritional state [21]; most of these fatty acids are bound to serum albumin while a small percent is unbound in the plasma. The amounts incorporated into plasma membranes (PM) of cells can be enhanced by up to 10 times through dietary supplements enriched in LCPUFA [22]–[24]. Further examination of the fatty acid composition in blood reported that roughly 5.3% of total serum fatty acids are LCPUFA (2.4% docosahexaenoic (DHA, C22∶6, n-3), 1.8% eicosapentaenoic (EPA, C20∶5, n-3), 1% docosapentaenoic (DPA, C22∶5, n-3 ) [25]; similar fatty acid concentration/composition has been reported in mice [26], [27].

It is speculated that due to its intrinsic ability to affect so many seemingly unrelated conditions, LCPUFA must function at a fundamental level common to most cells. There is strong evidence suggesting LCPUFA act by altering cell plasma membrane properties [28], [29] that affect GPCR function, however a direct interaction between LCPUFA and B_2_R has to be considered.

To gain insights into the mechanisms by which LCPUFAs influence endothelial cells, we have chosen to study B_2_R-dependent activation of the MAPK/ERK pathway, which is known to have an important role in the cardiovascular system [30]. In this study we tested if selected LCPUFAs can: (1) cause conformational changes in B_2_R, (2) activate ERKs via B_2_R, (3) modulate expression and activate eNOS; and (4) induce changes in expression of KLF2. Additionally we aimed to determine if a selective B_2_R antagonist can block EPA-mediated activation of B_2_R conformational changes and signaling. Furthermore, we investigated the dynamics of LCPUFA incorporation into cell membrane and changes in plasma membrane fluidity induced by LCPUFA.

## Results

### Detection of B2K-CC Conformational Change with LCPUFA

**Figure 1:**
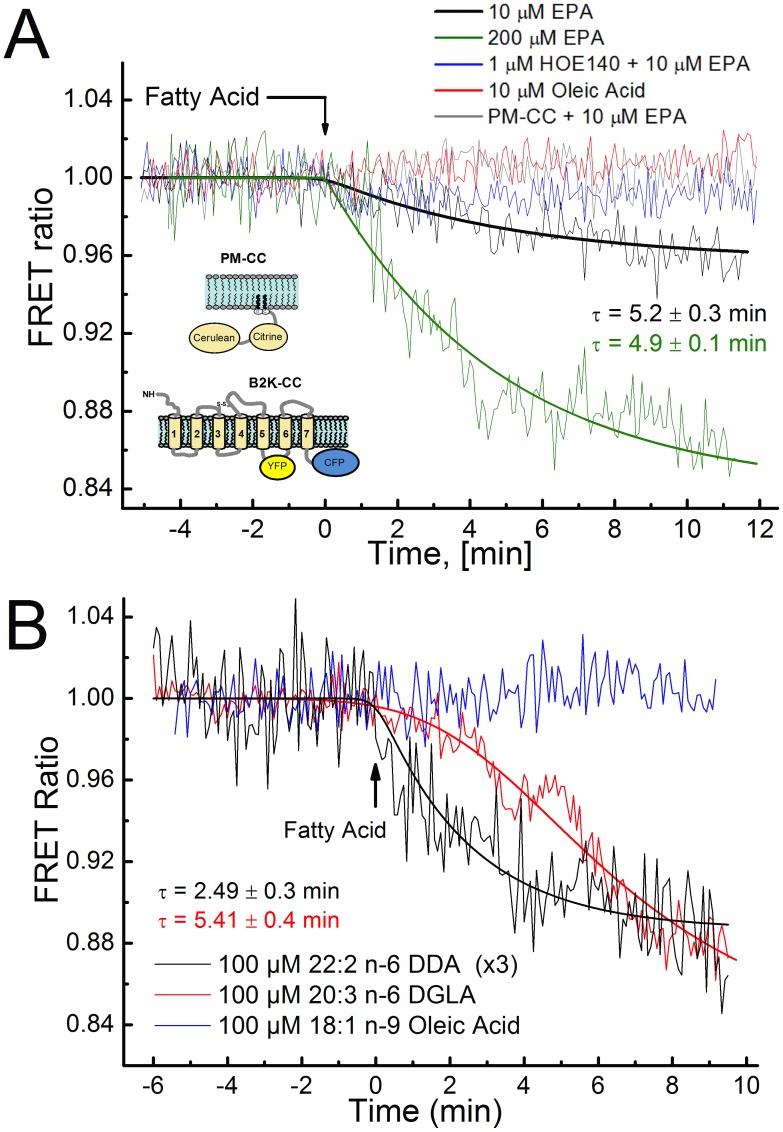
Response of B2K-CC FRET sensor and PM-CC (control FRET sensor) to stimulation by polyunsaturated fatty acids. B2K-CC transfected HEK-293 cells treated with (A) EPA, EPA and B_2_R inhibitor (HOE-140), oleic acid or (B) DDA, DGLA. Experiments were performed 24 h after transfection of B2K-CC FRET sensor or PM-CC in HEK293 cells on chambered cover glass at 37°C. FRET ratio was defined as ratio of Citrine emission intensity at 525 nm to Cerulean emission intensity at 475 nm.

To test if LCPUFAs induce conformational changes in B_2_R, we used the previously described B_2_R FRET sensor (B2K-CC) containing an intramolecular FRET pair that enables detection of conformational activity upon ligand stimulation [12]; a number of other GPCR FRET sensors has been constructed and used to study conformational GPCR activity using similar approach[9], [12], [31]–[36]. B2K-CC was expressed in human embryonic kidney 293 (HEK293) cells. In the presence of EPA, a decrease in the FRET ratio was observed with time constants (τ) of 4.9±0.1 min for 200 µM and 5.2±0.3 min for 10 µM treatments, respectively. In contrast Oleic acid had no effect on the B2K-CC FRET ratio (Fig. 1A). HEK293 cells expressing the control FRET sensor (PM-CC) showed no response to EPA (Fig. 1A). To test specificity of EPA-induced B_2_R conformational change, pre-incubation of the B2K-CC transfected HEK293 cells with the selective B_2_R antagonist, HOE-140 [37], blocked the conformational response of the B_2_R to EPA. Additionally, treatment with 100 µM of DDA (C22∶2, n-6) and DGLA (C20∶3, n-6) also caused a decrease in the FRET ratio observed with time constants of τ = 2.49±0.3 min and 5.41±0.4 min, respectively (Fig. 1B). Although GPCR FRET constructs of this type have been shown to have a reduced efficacy of cognate ligands in stimulating conformational changes (due to the insertion of fluorescing proteins into the native receptor structure [9], [38]), we were able to observe significant FRET response to LCPUFA.

### Fatty Acid Induced ERK Phosphorylation in HEK293 Cells Transfected with B_2_R

**Figure 2:**
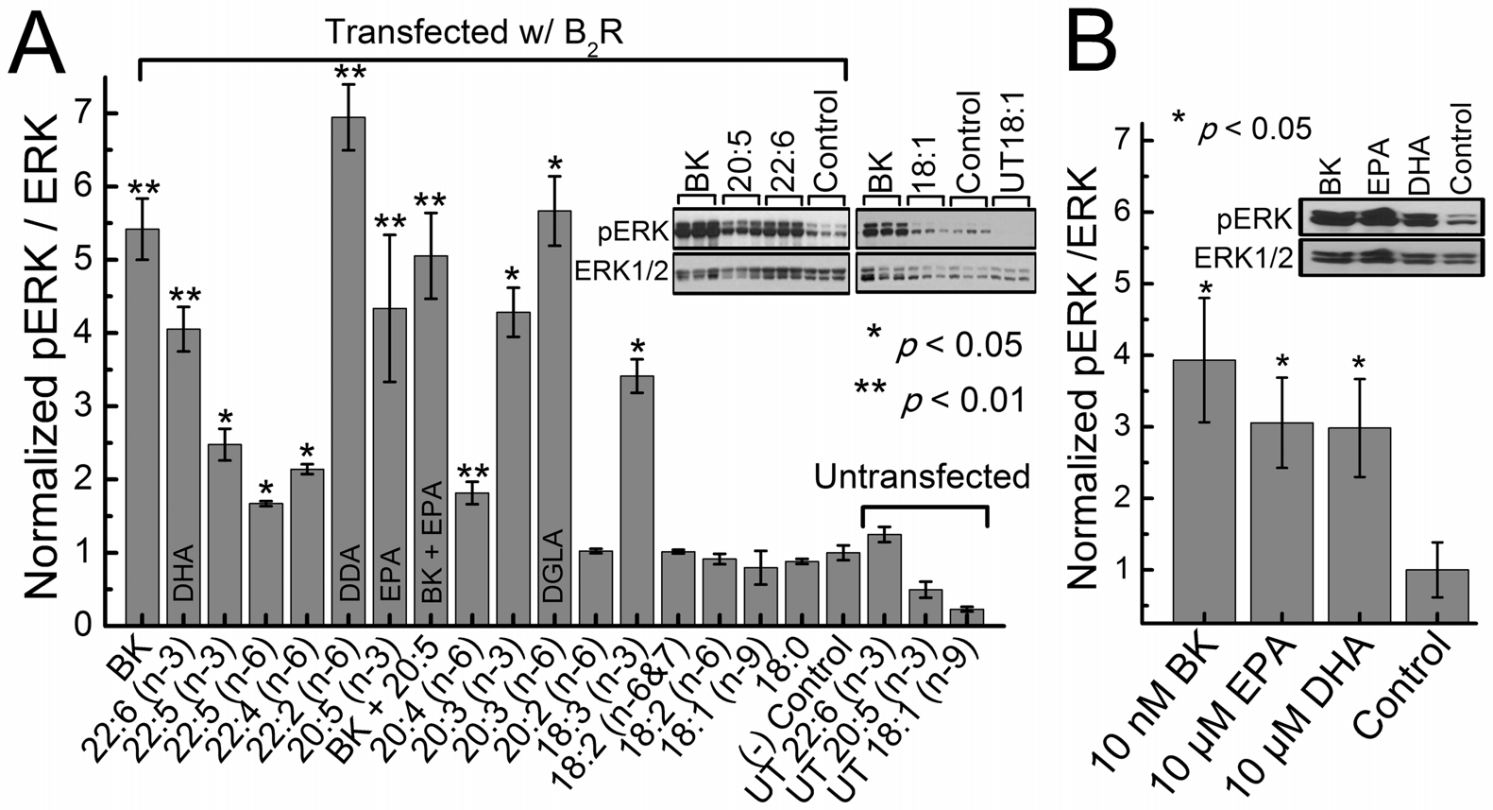
Activation of B_2_R by fatty acids in HEK293 cells. BK concentration in (A) was 100 nM (positive control) and (B) was 10 nM. Fatty acid concentrations were 10 µM in HEK293 cells transfected with B_2_R as described under Materials & Methods and treated for (A) 5 minutes or (B) 15 seconds in PBS at 37°C. Western blots were used to determine ERK1/2 phosphorylation. Data represent mean of n ≥ 6 experiments. Error bars indicate standard error of the mean (SEM).

To examine fatty acid activation of the ERK1/2 cascade via B_2_R, we treated B_2_R transfected HEK293 cells with distinct fatty acids for 5 minutes as shown on Fig. 2A. Untransfected cells were also treated with fatty acids since HEK293 express low levels of endogenous B_2_R [39], [40]; no significant increase in ERK1/2 phosphorylation was found compared to non-treated transfected cells, indicating that ERK1/2 activation was B_2_R dependent.

Note, that simultaneous treatment with BK (100 nM) and fatty acid (10 µM EPA) did not produce any additional increase in ERK phosphorylation beyond that caused by BK alone.

To exclude the possibility that LCPUFA metabolites are activating the ERK pathway, we treated B_2_R transfected HEK293 cells with EPA or DHA for 15 seconds (Fig. 2B). The short treatment significantly increased ERK1/2 phosphorylation but to a lower magnitude than after the 5 minute treatment; lower activation levels were also observed for the BK treated cells suggesting that the reduction in ERK activation after 15 second treatment is due to the intrinsic time scale needed for activation of ERK and not due to the finite production rate of LCPUFA metabolites. Furthermore, the majority of fatty acids have been reported to remain in the plasma membrane after 10 minutes [41] while the LCPUFA metabolizing enzyme, cyclooxygenase is localized in the endoplasmic reticulum [42] making it unlikely that the LCPUFAs metabolites are activating B_2_R in our experiments (in cells the prostaglandin production rate from EPA is of 4 to 5 hours [43], whereas in vitro the cyclooxygenase reaction kinetics with EPA at nanomolar concentrations is expected to be 7 minutes [44]).

### B_2_R Antagonist Blocks EPA Induced Activation of the ERK Cascade

**Figure 3:**
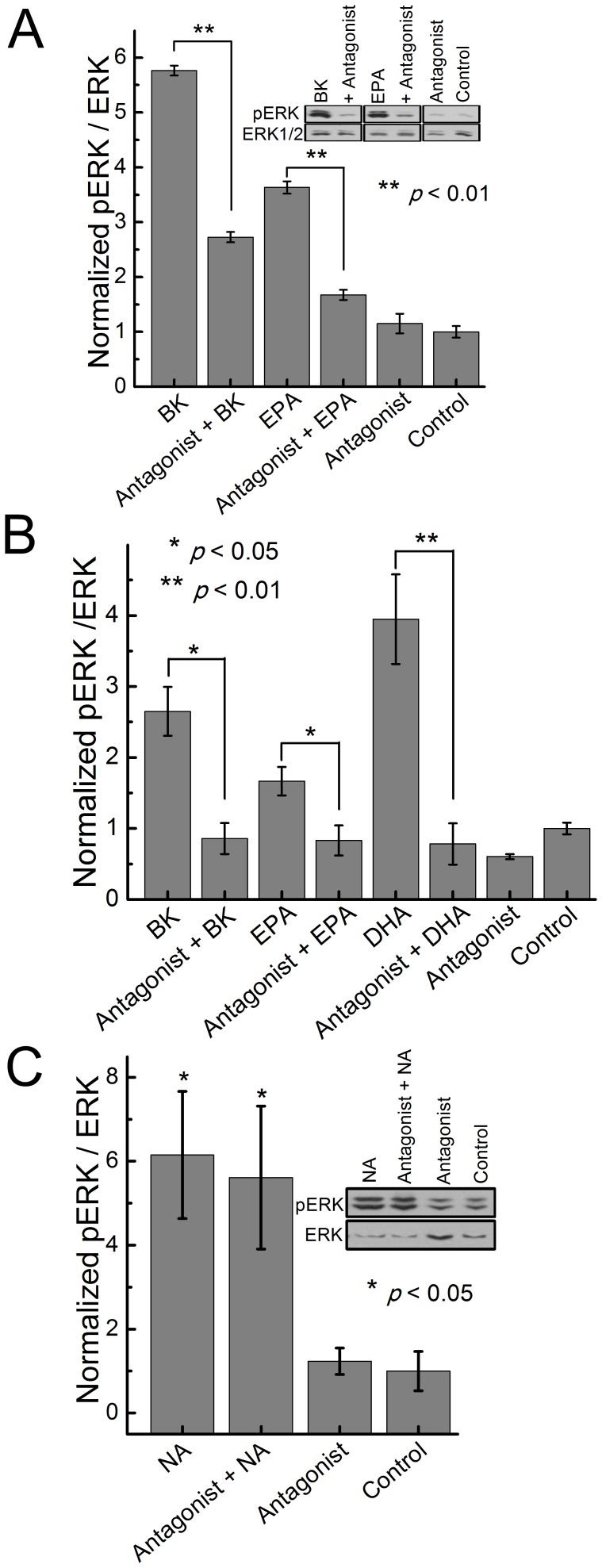
B_2_R antagonist inhibits ERK1/2 response to bradykinin and fatty acids. (A) Effect of 1µM of B_2_R antagonist HOE-140 on pERK stimulated with: 100 nM bradykinin, 1 µM EPA and 10 µM DHA in DPBS for 5 minutes at 37°C. Experiments were done 24 h after transfection of HEK293 cells with B_2_R in 12-well plates. (B) Effect of 1 µM of B_2_R antagonist HOE-140, on pERK stimulated with 10 nM BK, 1 µM EPA and 1 µM DHA in BAECs expressing endogenous B_2_R. Ratio of pERK to ERK was measured using western blots. (C) Effect of 1 µM of B_2_R antagonist HOE-140, on pERK stimulated with 100 nM NA in BAECs expressing endogenous adrenergic receptors. Data represent the mean of at least four independent experiments.

To determine if a B_2_R selective antagonist can inhibit fatty acid-induced ERK phosphorylation, HEK293 cells transfected with B_2_R were treated with EPA in the presence of B_2_R inhibitor, HOE-140 (Fig. 3A). Phospho-ERK immunoblot analysis showed that the antagonist is able to significantly inhibit ERK phosphorylation of cells treated with bradykinin (positive control) and EPA. To determine if these results were reproducible in a different cell line, we used bovine aortic endothelial cells (BAEC) expressing B_2_R at endogenous levels (no transfection was used). The cells were treated with BK (positive control), EPA, or DHA in the presence of the inhibitor. Phospho-ERK immunoblot analysis demonstrated that the inhibitor significantly blocked the ERK phosphorylation of the treated cells (Fig. 3B). To determine inhibitor specificity, we treated BAECs with noradrenaline (NA) to activate the endogenous adrenergic receptor and determine if the HOE-140 inhibitor would unspecifically inhibit ERK phosphorylation. The HOE-140 did not have any effect in inhibiting adrenergic receptor stimulation by NA (Fig. 3C) suggesting that HOE-140 selectively antagonizes B_2_ receptor.

### Measurement of Antagonist Potency of HOE-140 on B_2_R Expressed in HEK293 Cells

**Figure 4:**
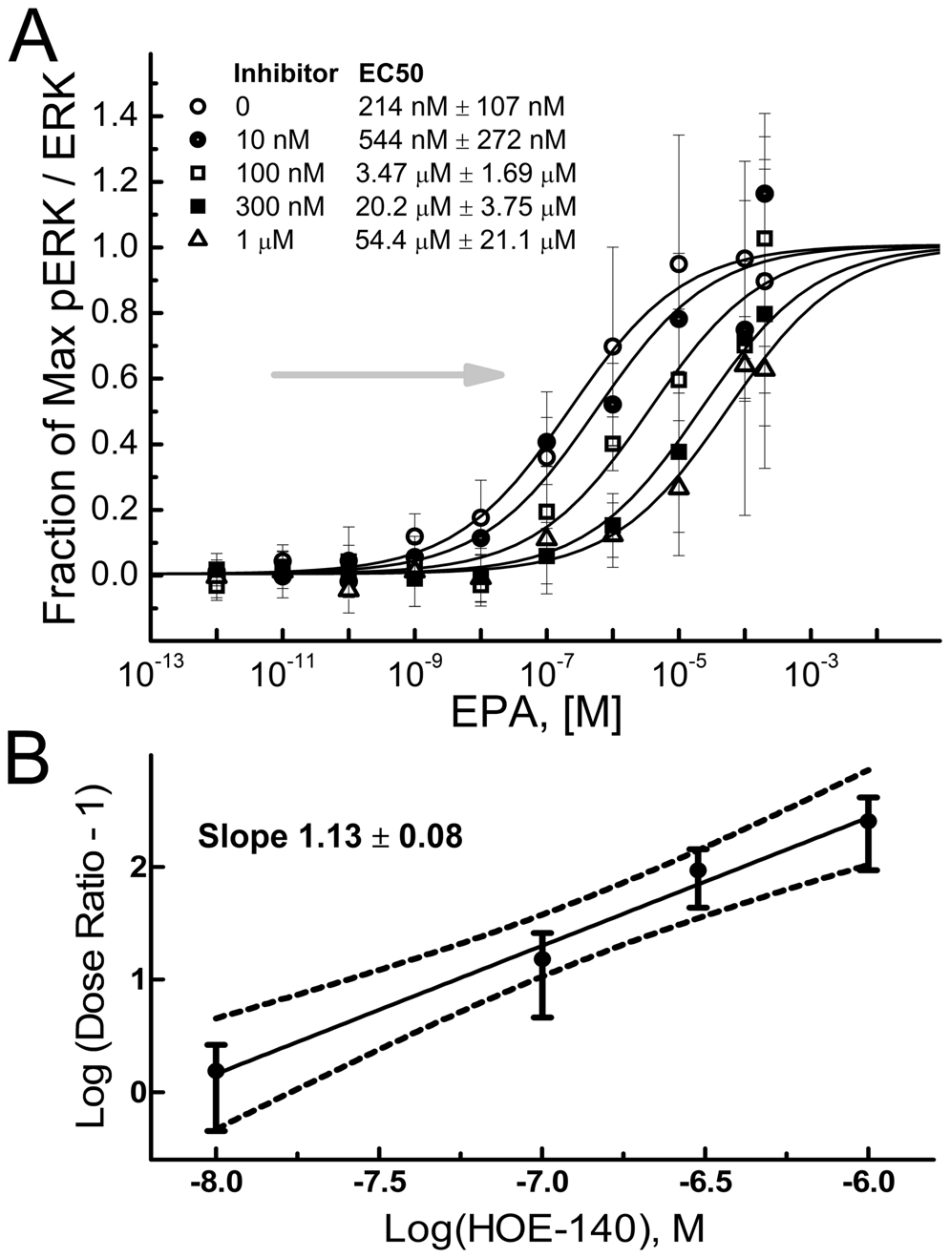
B_2_R antagonist shifts the EPA EC50 by inhibiting ERK1/2 response to fatty acids. (A) Dose response to EPA at different concentrations of antagonist HOE-140 in HEK293 cells. (B) Schild plot of HOE-140 antagonism on EPA-stimulated B_2_R. Experiments were done 24 h after transfection of HEK293 cells with B_2_R in 12-well plates. Ratio of pERK to ERK was measured using western blots. Data represent the mean of at least four independent experiments.

To further examine the mechanism of action of antagonist HOE-140 on inhibiting B_2_R activation by EPA, we have performed a Schild analysis of the antagonist-induced inhibition of ERK activation. HOE-140 produced a parallel rightward shift in the dependence of the B_2_R-mediated ERK activation on EPA concentration (Fig. 4A). The antagonist did not significantly affect the maximum possible effect (E_max_) of EPA and did not detectably affect ERK phosphorylation in the absence of the agonist (Fig. 3). The Schild slope was 1.13±0.08 (Fig. 4B). These observations suggest that HOE-140 acts as a competitive antagonist of EPA stimulated ERK phosphorylation at the B_2_R over the range of antagonist concentrations tested.

### B_2_R Antagonist Blocks EPA-induced Activation and Expression of eNOS

**Figure 5:**
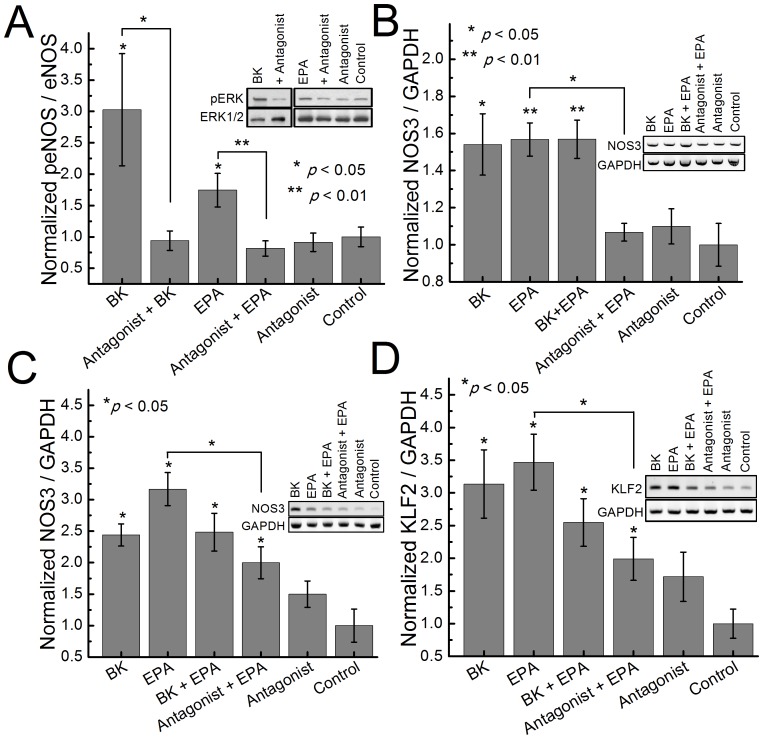
Fatty acids cause eNOS activation and alters NOS3 and KLF2 expression. (A) Effect of 1 µM of B_2_R antagonist HOE-140 on peNOS stimulated with: 10 nM bradykinin or 1 µM EPA on BAECs in DPBS for 5 minutes at 37°C. Effect of 1 µM of B_2_R antagonist HOE-140 on NOS3 expression with 10 nM BK or 1 µM EPA (B) BAECs and (C) HCAECs DPBS for 1 hour at 37°C. (D) Effect of 1 µM of B_2_R antagonist HOE-140 on KLF2 expression with 10 nM bradykinin or 1 µM EPA. Data represent the mean of at least four independent experiments.

To investigate if EPA-dependent B_2_R activation has a direct biological effect, we investigated eNOS activation via phosphorylation at serine 1177 which is known to activate the eNOS enzyme leading to production of nitric oxide (NO) [45]. Treatment of BAECs with BK or EPA for 5 minutes induced an increase in eNOS phosphorylation (Fig. 5A). To determine if eNOS activation is a result of EPA-induced B_2_R activation, we repeated the experiment in the presence of the antagonist HOE-140. The antagonist blocked the B_2_R activation by BK and EPA.

### NOS3 Expression is Modulated by EPA-dependent B_2_R Activation

To further characterize the association between the fatty acids, B_2_R and eNOS activation, alterations in NOS3 gene expression were studied. BAECs were treated with BK, EPA, or both for 1 hour prior to gene expression analysis via RT-PCR. NOS3 expression levels were increased as a result of BK or EPA stimulation while simultaneous treatment with BK and EPA did not increase the expression further (Fig. 5B). Specificity to B_2_R stimulation was determined by the ability of the B_2_R antagonist, HOE-140, to significantly inhibit the increase in expression. To support these findings, the experiment was repeated in human coronary artery endothelial cells (HCAEC) and similar results were obtained (Fig. 5C).

### Expression of NOS3 Transcription Factor, KLF2, is Modulated by EPA-dependent B_2_R Activation

To gain insights into the possible upstream regulation of EPA-induced B_2_R stimulation that causes alterations in NOS3 expression, we investigated expression of Kruppel-like factor 2 (KLF2; which is a known transcription factor that induces eNOS expression [46]). Treatment of BAECs with BK or EPA alone, or in combination for 1 hour was performed prior to analysis of gene expression via RT-PCR. KLF2 expression levels were increased as a result of BK or EPA stimulation while simultaneous treatment with both did not increase the expression further (Fig. 5D). B_2_R dependent regulation of KLF2 expression was confirmed by the ability of the selective B_2_R antagonist to significantly inhibit the expression increase.

### EPA Increases Plasma Membrane Fluidity of HEK293 Cells

**Figure 6:**
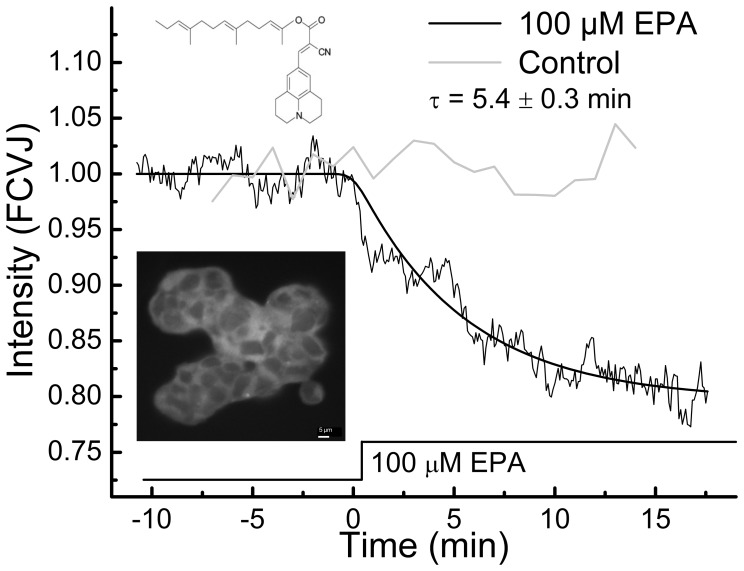
Membrane viscosity changes with fatty acids. Measured signal intensity of HEK293 cells stained with FCVJ as a response to 100 µM EPA or control treatment in chambered cover glass at 37°C. Data represents the mean of at least 12 independent experiments.

To gain insights into possible non-specific mechanisms by which LCPUFA is activating the B_2_R and to determine how fast EPA partitions into the cell membrane, we investigated effects of fatty acids on membrane viscosity. Using the molecular rotor farnesyl-(2-carboxy-2-cyanovinyl)-julolidine (FCVJ) and picosecond time-resolved fluorescent microscopy, we recorded time-dependent changes in membrane fluidity in response to EPA treatment. Since FCVJ has a viscosity-dependent quantum yield [47], we stained HEK293 cells with FCVJ and recorded its signal intensity. Treatment with 100 µM of EPA caused a time-dependent decrease in fluorescence intensity compared to the control solution, indicating an increase in membrane fluidity with a time scale of 5.4±0.3 min (Fig. 6). A treatment with control solution of 0.1% Ethanol and 3.5×10^−5%^ BSA in DPBS did not induce significant changes in fluidity.

## Discussion

Analysis of the FRET data (Fig. 1) indicates that EPA-treated B_2_R FRET sensor undergoes the conformational change (negative change in FRET ratio) that is similar to the one induced by its cognate ligand, BK [12] but occurs on a longer time scale. We have previously shown that the time scale of B2K-CC FRET sensor response to BK stimulation is a few seconds (depending on the concentration) [12]. In contrast Fig. 1 shows that LCPUFA induce conformational changes in B_2_R on a time scale of 2 to 5 minutes. Note, that the time scale of the B2K-CC response to LCPUFA is similar to the time scale of LCPUFA incorporation into the membrane bilayer (see below). The kinetics of FRET response (Fig. 2B) is expected to be different between DDA and DGLA due to differences in the structure of these fatty acids. Compared to the untreated receptor, our data show that EPA, DDA, and DGLA induce a decrease in FRET signal that ultimately saturates and stabilizes at a different FRET level, indicating that B_2_R changes conformational state (Fig. 1).

The maximal effect (E_max_) of fatty acids on ERK1/2 phosphorylation is lower as compared to stimulation by BK (Fig. 1A, Fig. 2A,B) suggesting that the interactions with fatty acids stabilize the receptor in an activated state that is different from the one stimulated by BK. Although our primary focus in this study was EPA, other LCPUFAs, both n-3 and n-6, also caused a significant, B_2_R-dependent ERK phosphorylation with varying potency (Fig. 2A). To decipher a possible trend in fatty acid induced B_2_R-dependent ERK activation, we tested fatty acids with different alkyl chain lengths, saturation level and positions of double bonds. Our data presented in Fig. 2A suggest that the length of the fatty acid and the number of carbon chains play a major role in B_2_R activation. More specifically, our data suggest that significant B_2_R activation requires fatty acids to have an alkyl chain length of no less than 20 carbons with 3 or more double bonds, except for alpha-linolenic acid (18∶3 n-3) which showed significant potency in ERK activation. Note, EPA appears more potent in the FRET assay than in the ERK assay due to differences in treatment concentrations. Overall, our results are similar to those we earlier observed for the PTH1 receptor [1] and consistent with other studies in which specific GPCRs have been shown to become activated by fatty acids in a length-dependent manner [48]–[51].

The slope of the Schild plot [52], [53] (Fig. 4B) suggests that EPA is interacting with the orthosteric binding site of B_2_R. Note, however, that the concentration of fatty acids in the membrane are expected to be significantly higher than concentrations in the suspension buffer due to natural tendency of LCPUFA to partition into bilayers [28]. The lipid bilayer effectively increases the concentration of LCPUFA in GPCR environment thereby increasing the probability of their interaction. In contrast to our earlier report on PTH1 receptor [1] we did not observe synergism from simultaneous stimulation by BK and LCPUFA (Fig. 2 A, 5 B, 5 C) supporting the hypothesis that EPA acts through the orthosteric site of B_2_R. However due to the high concentrations of LCPUFA in the membrane, contributions from allosteric and nonspecific interactions cannot be excluded.

BK is a potent stimulator of endothelial nitric-oxide synthase (eNOS), the enzyme that catalyze the production of nitric oxide (NO) via B_2_R signaling pathway [13]. NO contributes to vessel homeostasis by inhibiting vascular smooth muscle contraction and growth, platelet aggregation, and leukocyte adhesion to the endothelium [14]. In our study, EPA had similar biological effects on eNOS as BK. EPA was able to increase phosphorylation of serine 1177 which activates the eNOS enzyme to produce nitric oxide (NO) [45] in a B_2_R-dependent manner. According to a recent study, EPA stimulates phosphorylation of AMP-activated protein kinase (AMPK)-Thr172 leading to an increase in eNOS phosphorylation and NO release [54], while our present results similarly show that EPA increases B_2_R-dependent expression and activation of eNOS. NO plays an important role in the protection against the onset and progression of cardiovascular disease [14] and this finding provides new insights into the putative mechanism by which LCPUFAs act at preventing and alleviating CVDs; these findings are in agreement with previous studies that reported EPA-induced NO production in endothelial cells [19], [20].

KLF2 is an endothelial transcription factor that is uniquely induced by fluid shear stress [55]. Blood flow turbulence at vessel bifurcations and curvatures have been implicated as a factor in atherosclerosis [56]. The ability of LCPUFA to increase expression levels of KLF2 (Fig. 5 D), which was the first endothelial transcription factor shown to be uniquely induced by flow, might provide a molecular basis for beneficial effects of LCPUFA; notably it has been recently reported that LCPUFA supplemented diet plays a similar role in preventing bone loss as mechanical loading of bone (which is known to be fluid shear stress dependent) [57].

Changes in membrane-dependent functions of receptors and membrane associated proteins have been hypothesized to depend on cell membrane fluidity [9], [10], [12], [47], [58]. It has been shown that stimulation with BK decreases cell membrane fluidity [59] which is in contrast to the fluidizing effect of EPA on the cell membrane; therefore LCPUFA-induced changes in membrane fluidity are unlikely to be responsible for the observed response of B_2_R to LCPUFA. The time scale of LCPUFA incorporation into membrane (Fig. 6) is within the same range as that one of EPA-induced conformational changes in both B_2_R and PTH1R [1]. This finding suggest that fatty acids first partition into the cell membrane and then may either specifically bind to the transmembrane/intracellular domain of the GPCR or affect membrane properties in a non-specific way, stabilizing GPCR in a tertiary conformation required for efficient signaling. As an example of the effects of other membrane components, several GPCR structure determination efforts have shown that the addition of cholesterol analogs is often critical for maintaining protein stability [60]. The PTH1R and B_2_R show a similar change (decrease) in FRET in response to LCPUFA that may be suggestive of a general underlying mechanism. However, the existence of putative general mechanism by which GPCRs are activated by LCPUFA does not diminish the relevance to hemodynamic function or function in other biological tissues because cells in specialized tissues express specific GPCRs at relatively high levels as e.g. B_2_R in endothelial cells [61]; as noted above activation of B_2_R in endothelial cells is known to invoke a number of physiological hemodynamic responses.

While bradykinin treatment favors cardioprotective properties, continuous treatment induces side effects [11]. A similar profile has not yet been reported for EPA or other LCPUFAs. In contrast to bradykinin, one possible explanation for the differences in activity is the ability of EPA to alter other GPCRs that serve other functions. For example, Grp120, a GPCR that binds to EPA, mediates potent anti-inflammatory and insulin sensitizing effects [62]. EPA may also bind to other GPCRs important in the prevention of CVDs.

Currently, therapeutic medications are available for the prevention and/or treatment of CVDs but are expensive and have significant side effects. Studies have shown that supplementation with n-3 LCPUFA after myocardial infarction reduced the risk of recurrence, stroke, and death by 10% [63]. Many studies suggest n-3 LCPUFAs, derived from seafood and plant sources may reduce CVD risk [64], [65]. n-3 fatty acids have been reported to contain antihypertensive effects, reducing features of inflammatory atherosclerotic plaques, and decrease in death rate over a two year post myocardial infarction [66]. The importance of BK signaling in functioning of cardiovascular system and the effects of LCPUFA on BK pathway described in this study call for more detailed studies of the role of LCPUFA on BK pathway. Further studies in both in in vitro and in vivo models may contribute to development of alternative approaches for CVD treatments.

Overall, we characterized the effect of LCPUFA on the B_2_R and our results indicate that EPA is a novel B_2_R agonist. More specifically, we demonstrated the ability of LCPUFA (EPA, DHA, DDA, or DGLA) to act as agonist to the B_2_R at nanomolar concentrations and to induce receptor conformational changes. EPA was able to induce a B_2_R response with an EC50 value of 214 nM ±107 nM. The Schild analysis indicated that EPA was competitively binding to the orthosteric site of the B_2_R. Furthermore, we showed that other LCPUFAs were also able to activate B_2_R at the concentrations of fatty acids typically found in blood. Experiments to determine activation of downstream signaling pathways revealed an increase in ERK phosphorylation, regulation of eNOS activity and expression. Collectively, these results provide a putative molecular basis for the involvement of the B_2_R in mediation of LCPUFAs effects in endothelial cells. We believe our study is a starting point for more detailed investigations of interactions between fatty acids and other types of GPCRs.

## Materials and Methods

### Cell Culture, Transfection, and Chemicals

HEK293 (American Type Tissue Collection, passages 2–10) cells were grown in DMEM media (Invitrogen, Carlsbad, CA, USA) containing 4.5 g/L D-Glucose and transfected using Targefect-293 (Targeting Systems, Santee, CA, USA). Primary human coronary artery endothelial cells (HCAECs) and bovine aeortic endothelial cells (BAECs) were purchased from Lonza Walkersville (Walkersville, MD) and Cell Applications (San Diego, CA) and grown in complete EC growth media (EGM-2, Lonza). Human bradykinin B_2_R was obtained from the University of Missouri cDNA Resource Center (Rolla, MO). HOE-140 was purchased from Enzo (Plymouth Meeting, PA, USA). Adrenic Acid 22∶4 (n-6) was purchased from Enzo (Plymouth Meeting, PA, USA). Docosahexaenoic Acid 22∶6 (n-3) and Eicosapentaenoic Acid 20∶5 (n-3) were obtained from Cayman Chemical (Ann Arbor, MI, USA). Dihomo-gamma-Linolenic acid 20∶3 (n-6), Docosadienoic Acid 22∶2 (n-6), Docosapentaenoic Acid 22∶5 (n-3), Docosapentaenoic Acid 22∶5 (n-6), and Eicosatrienoic Acid 20∶3 (n-3) were purchased from Nu-Chek-Prep (Elysian, MN, USA). Arachidonic Acid 20∶4 (n-6), Conjugated Linoleic Acid 18∶2 (n-6/7), and Eicosadienoic Acid 20∶2 (n-6), were purchased from Matreya (Pleasant Gap, PA, USA) alpha-Linolenic acid 18∶3 (n-3), Linoleic Acid 18∶2 (n-6), Oleic Acid 18∶1 (n-9), and Stearic Acid 18∶0 were purchased from Sigma-Aldrich (St. Louis, MO, USA). GF109203X and H-89 were purchased from Enzo (Plymouth Meeting, PA, USA).

### Fatty Acid and Inhibitor Treatment

To examine fatty acid dependent ERK phosphorylation, HEK293 cells transfected with B_2_R were treated with various fatty acids at 10 µM concentration containing 0.1% Ethanol and 3.5×10^−5^% BSA in DPBS at 37°C for 5 minutes. The negative control treatment consisted of only 0.1% Ethanol and 3.5×10^−5^% BSA in DPBS at 37°C for 5 minutes. To characterize the effect of B_2_R inhibitor on fatty acid stimulation, HEK293 cells transfected with B_2_R were incubated with either 1 µM, 300 nm, 100 nm, or 10 nm of the B_2_R inhibitor, HOE-140, for 30 minutes prior to fatty acid treatment.

### Plasma Membrane Localized FRET Sensor (PM-CC)

A control FRET sensor that is localized to the plasma membrane of cells (PM-CC) was constructed as previously described [67]. The construct PM-CC encodes a protein that is comprised of Citrine and Cerulean linked together with a short and flexible GGGGPV (ProVal to encode Age1 restriction site) linker peptide to ensure Förster resonance energy transfer between the two fluorescent proteins, and is fused to another 10 residue leader peptide MGCINSKRKD to direct its translocation to plasma membrane [68].

### B_2_R FRET Sensor (B2K-CC)

B2K-CC was constructed and characterized as described in our previous study [69]. A primer extension method was used to construct B2K-CC by inserting EYFP into the third cytoplasmic loop at a position between F232 and K233 and fusing ECFP to the truncated C terminus at the position C329. The sequences of all constructs were confirmed by sequencing service.

### FRET Measurements

FRET measurements in single living cells, 24 hours after being transfected, were performed using multichannel time resolved single photon counting as previously described [69]. Briefly, fluorescence emission kinetics and spectra were measured by using a multichannel, time-correlated single photon counting spectrograph (PML-16/SPC630; Becker & Hickl, Berlin, Germany) coupled to an inverted microscope (Axiovert 200 M; Zeiss, Thornwood, NY) via fiber optic link. A femtosecond Ti:Sapphire oscillator (Spectra-Physics, Irvine, CA, USA) was used as the excitation source. The repetition frequency of the light pulses from the oscillator was reduced to 8 MHz and the wavelength was doubled to 435 nm. The excitation light was defocused to a spot size of 20–50 µm to enable spatially homogenous excitation of a single cell. Single cell fluorescence spectra were obtained by integrating time-resolved fluorescence data. Presented data were recorded by detecting fluorescence emission polarized at the magic angle (54.7°) to the polarization of the excitation light at 435 nm to reduce contributions from potential reorientation of the sensor in the plasma membrane under shear stress. FRET ratio was defined as ratio of Citrine emission intensity at ∼ 525 nm to Cerulean emission intensity at 475 nm. Duration FRET measurements were kept to a minimum to prevent photobleaching.

### Western Blot Analysis

HEK293 cells were washed twice in DPBS, collected, and lysed in lysis buffer containing 50 mM Tris –HCl, 135 mM NaCl, 60 mM n-octyl beta-D-glucopyranoside (EMD Chemicals, Gibbstown, NJ, USA), protease inhibitor cocktail, and phospahtase inhibitor cocktail (Roche, Basel, Switzerland) for 30 minutes on ice. SDS sample buffer was added to the lysate and incubated at 95°C for 5 min. Cell extracts were resolved by SDS-PAGE and transferred to polyvinylidine difluoride (PVDF) membranes. Blots were probed with anti-phospho-p44/42 MAPK (Erk1/2 rabbit polyclonal), anti-p44/42 MAPK (Erk1/2 rabbit polyclonal; Cell Signaling Technology, Beverly, MA, USA), p-NOS3 (Ser 1177; sc-12972 rabbit polyclonal), and NOS3 (N-20; sc-653 rabbit polyclonal; Santa Cruz Biotechnology, Santa Cruz, CA, USA). Immunoreactive bands were detected with the appropriate horseradish peroxidase-conjugated secondary antibodies (anti-rabbit; Cell Signaling Technology, Beverly, MA, USA, 7074) and visualized by enhanced chemiluminescence (Thermo Scientific, Rockford, IL, USA). The band intensities were measured by densitometry analysis using Image J (NIH, Bethesda, MD, USA) and the increment of phosphorylated ERK in HEK293 cells overexpressing B_2_R was calculated as the pERK/ERK ratio.

### Semi-quantitative RT-PCR

Semi-quantitative reverse transcription PCR (RT-PCR) was performed using the Perltier Thermal Cycler PTC-225. RNA from each cell line was extracted and purified using the TRIzol reagent (Life Technologies, Carlsbad, CA, USA) according to the manufacturer’s instructions. RNA was measured spectrophotometrically at 260 and 280 nm to assess quantity and quality. For each sample, 1 µg of RNA were transcribed using the RNA to cDNA Premix (Oligo dT) kit (Clontech, Mountain View, CA, USA) for 1 h at 42°C, then at 70°C for 10 min to stop the reaction. Primers for specific detection of nitric oxide synthase 3 (NOS3) were: (ENOS-F: 5′- GTCCTGCAGACGGTGCAGC-3′; ENOS-R: 5′- GGCTGTTGGTGTCTGAGCCG-3′). The kruppel-like factor 2 (KLF2) were: (KLF2 648-F: 5′- CCCAGCCTTCGGTCTCTT-3′; KLF2 911-R: 5′-CAGTGGTAGGGCTTCTCACC-3′). The glyceroldehyde 3-phosphate dehydrogenase gene (GAPDH) was used as the internal standard. Primers for GAPDH were used for normalization (GAPDH-F: 5′-AAATCCCATCACCATCTT-3′; GAPDH-R: 5′-TTCCACGATACCAAAGTT-3′). PCR products were separated on 1.5% agarose gels and stained with GelRed nucleic acid stain (Phenix research products, Candler, NC, USA).

### FCVJ Staining

A staining solution was prepared from 50 µl stock solution of 20 mM of the FCVJ in dimethyl sufoxide (DMSO), which was dispersed in 2 ml fetal cow serum under vigorous stirring as described [47]. 10 ml of DMEM was added. The slides with the cells were covered with the staining solution. Incubation took place over 10 min in the dark at 37°C. After the incubation period, the cells were rinsed with HBSS, and the fluorescence checked under an epifluorescent microscope (Diaphot TMD, Nikon, Garden City, NY, USA) using the G2B filter set.

### Statistical Analyses

Statistical significance was evaluated for differences between groups from at least three independent experiments using Stu dent’s *t*-test. A *p* value of <0.05 was considered to be statistically significant. Calculations of the mean, standard error of the mean, and Student’s *t*-test were performed using Microsoft Excel and OriginPro (OriginLab Co., Northampton, MA).
